# Identification of ESM1 overexpressed in head and neck squamous cell carcinoma

**DOI:** 10.1186/s12935-019-0833-y

**Published:** 2019-05-02

**Authors:** Hongbo Xu, Xiaohong Chen, Zhigang Huang

**Affiliations:** Department of Otolaryngology-Head and Neck Surgery, Key Laboratory of Otolaryngology Head and Neck Surgery, Beijing Tongren Hospital, Capital Medical University, Beijing, 100730 China

**Keywords:** ESM1, Head and neck cancer, Regulation

## Abstract

**Background:**

Endocan, also known as endothelial cell specific molecule-1 (ESM1), is a 50 kDa soluble proteoglycan which is frequently overexpressed in many cancer types. Whether it is dysregulated in head and neck squamous cell carcinoma (HNSCC) has not been investigated.

**Methods:**

We analyzed the expression of ESM1 using bioinformatics analysis based on data from The Cancer Genome Atlas (TCGA), and then validated that ESM1 was significantly overexpressed in human HNSCC at the protein level using immunohistochemistry. We also analyzed the genes co-expressed with ESM1 in HNSCC.

**Results:**

The most correlated gene was angiopoietin-2 (ANGPT2), a molecule which regulates physiological and pathological angiogenesis. Several transcription factor binding motifs including SMAD3, SMAD4, SOX3, SOX4, HIF2A and AP-1 components were significantly enriched in the promoter regions of the genes co-expressed with ESM1. Further analysis based on ChIP-seq data from the ENCODE (Encyclopedia of DNA Elements) project revealed that AP-1 is an important regulator of ESM1 expression.

**Conclusions:**

Our results revealed a dysregulation of ESM1 and a potential regulatory mechanism for the co-expression network in HNSCC.

**Electronic supplementary material:**

The online version of this article (10.1186/s12935-019-0833-y) contains supplementary material, which is available to authorized users.

## Background

Head and neck squamous cell carcinoma (HNSCC) includes many cancers in the head and neck originating from a variety of sub-sites including the lip, oral cavity, nasopharynx, oropharynx, and larynx. HNSCC is the sixth most common cancer worldwide. There are about 650,000 new cases and nearly 350,000 patient deaths from HNSCC annually [1]. The most common causes include tobacco and alcohol consumption, but human papilloma virus (HPV) has been shown to be a primary cause of oropharyngeal cancers [2]. Our understanding of the molecular and genetic abnormalities leading to oncogenesis of HNSCC has greatly increased over the past decade. Many studies based on genomic and expression profiles have provided a more thorough understanding of the molecular abnormalities in head and neck cancer to help guide the development of new therapeutic agents [3]. For example, mutational analysis has revealed that many genes such as *TP53*, *CDKN2A*, *PTEN*, *PIK3CA*, *HRAS*, *NOTCH1*, *IRF6*, and *TP63* are frequently mutated in HNSCC [4]. As for gene expression, many genes, such as βIII-tubulin (TUBB3) [5], TMEM16A/ANO1 [6], homeobox gene family (HOX) members [7] and metalloproteinases (MMPs) [8], have been found to be dysregulated in HNSCC. It is crucial to investigate novel molecular mechanisms involved in proliferation, apoptosis, and invasion of HNSCC and provide effective biomarkers or drug targets for diagnosis and prevention of the disease.

Endocan, also called endothelial cell specific molecule-1 (ESM-1), is an endothelial cell-associated proteoglycan [9]. It is up-regulated by pro-inflammatory cytokines, such as tumor necrosis factor-α (TNF-α), interleukin (IL)-1 and microbial lipopolysaccharide, as well as by proangiogenic molecules such as vascular endothelial growth factor (VEGF) [10]. ESM1 is possibly involved in neoangiogenesis, and as a promising biomarker of endothelial dysfunction and inflammation, it has being increasingly studied in recent years in a wide spectrum of healthy and pathophysiological processes [11–14]. ESM1 is preferentially expressed in tumor endothelium [15], and is dramatically overexpressed in many cancers including non-small cell lung cancer [16], colorectal cancer [17], clear cell renal cell carcinoma [18], gastric cancer [19], hepatocellular carcinoma [20], pituitary adenoma [21], ovarian cancer [22], and brain cancers [23]. In addition, serum endocan was reported to be a potential marker for cancer diagnosis and prognosis [19, 24–28]. Therefore, ESM-1 may be useful as a therapeutic cancer target.

The differential expression of ESM1 has not been investigated in HNSCC. In this study, we analyzed the expression of ESM1 in cancerous and adjacent normal HNSCC tissue using RNA-seq data from The Cancer Genome Atlas (TCGA) [29], and we used immunohistochemistry to examine whether ESM1 was overexpressed at the protein level in HNSCC tissue. We also identified a set of genes co-expressed with ESM1, and found that transcription factor binding motifs including SMAD3, SMAD4, SOX3, SOX4, HIF2A and AP-1 components were significantly enriched in the promoter regions of these correlated genes. We further confirmed reliable motifs using ChIP-seq data from the ENCODE (Encyclopedia of DNA Elements) project via the University of California, Santa Cruz (UCSC) genome browser [30]. Our results show that AP-1 plays an important role in the regulation of ESM1 expression, and provide important functional clues about ESM1 dysregulation and its regulatory mechanism in HNSCC.

## Materials and methods

### Data set

The Cancer Genome Atlas (TCGA) data related to HNSCC were downloaded from Xena public data hubs (http://xena.ucsc.edu/). In the UCSC-hosted database, TCGA data sets are normalized and can be explored and downloaded.

TCGA copy number profile was measured experimentally using whole genome microarray. Gene-level copy number variation (CNV) was estimated using the GISTIC2 method [31]. GISTIC2 further thresholded the estimated values to − 2, − 1, 0, 1, 2, representing homozygous deletion, single copy deletion, diploid normal copy, low-level copy number amplification, or high-level copy number amplification.

The BioXpress database, which also uses TCGA data, was used to query differential expression [32].

### Samples and immunohistochemical analysis

After informed consent had been obtained, all specimens were collected from patients. Twenty-one cases of laryngeal or hypopharyngeal squamous cell carcinoma were studied. Paraffin embedded cancer tissue and peri-cancerous tissue were selected for the immunohistochemical tests. After dehydration, transparent, paraffin embedded, frozen tissues were made into 2 μm serial sections. Slides of tissue were incubated for 40 min at 70 °C, rehydrated in alcohol solution, and then washed with water. Then the slides were treated with 3% H_2_O_2_ for 10 min, and then EDTA pH 9.0 for 1 min 50 s. For immunohistochemical analysis, the slides were incubated with anti-ESM1 (ab56914, Abcam, Cambridge, England) (1:300) for 1 h at 37 °C. After thorough washing with PBS, the slides were incubated with horseradish peroxidase (HRP) conjugated anti-rabbit IgG at 37 °C for 15 min, and then thoroughly washed again. After washing, bound antibody was detected using the 3,3′-diaminobenzidine (DAB) reaction. Nuclear counterstaining was performed with hematoxylin. Control sections were subjected to the same procedure except that the first antibody was eliminated from the incubation. Positive staining was seen as a brown color of varying intensity, and a positivity score was assigned for statistical analysis (Chi squared test).

### Immunofluorescence assay

For immunofluorescence staining of ESM1 and ANGPT2, paraffin-embedded 3 μm serial sections of five cases of laryngeal or hypopharyngeal squamous cell carcinoma samples were deparaffinized and rehydrated. Preheat EDTA 8.0 was used for repairing in the high pressure cooker. Polyclonal rabbit anti-human primary antibodies anti-ESM1/FITC (ab103590, Abcam, Cambridge, England) and anti-ANGPT2/TRITC (Abcam, Cambridge, England) (1:100) were applied overnight at 4 °C. After washing, fluorescently conjugated secondary antibodies were used. Nuclear counterstain was achieved using DAPI staining. All fluorescently stained images were taken using an Olympus BX-51 upright light microscope (Olympus, Tokyo, Japan). Each site was imaged in all channels and overlaid in DPViewer version before examination in Photoshop.

### Transcription factor binding motifs

The HOMER (Hypergeometric Optimization of Motif EnRichment) program package (v4.9, http://homer.ucsd.edu/) [33] was used for transcription factor binding motif analysis according to the procedure in the online guide. The region – 500 bp to + 100 bp from the transcription start site (TSS) in gene sets of interest was searched for enriched motifs against random background regions using the findMotifs.pl program. Enriched motifs were further validated by ChIP-seq data integrated in the transcription factor ChIP-seq (161 factors) track on the UCSC genome browser (http://genome.ucsc.edu).

## Results

### ESM1 is overexpressed in HNSCC

**Fig. 1:**
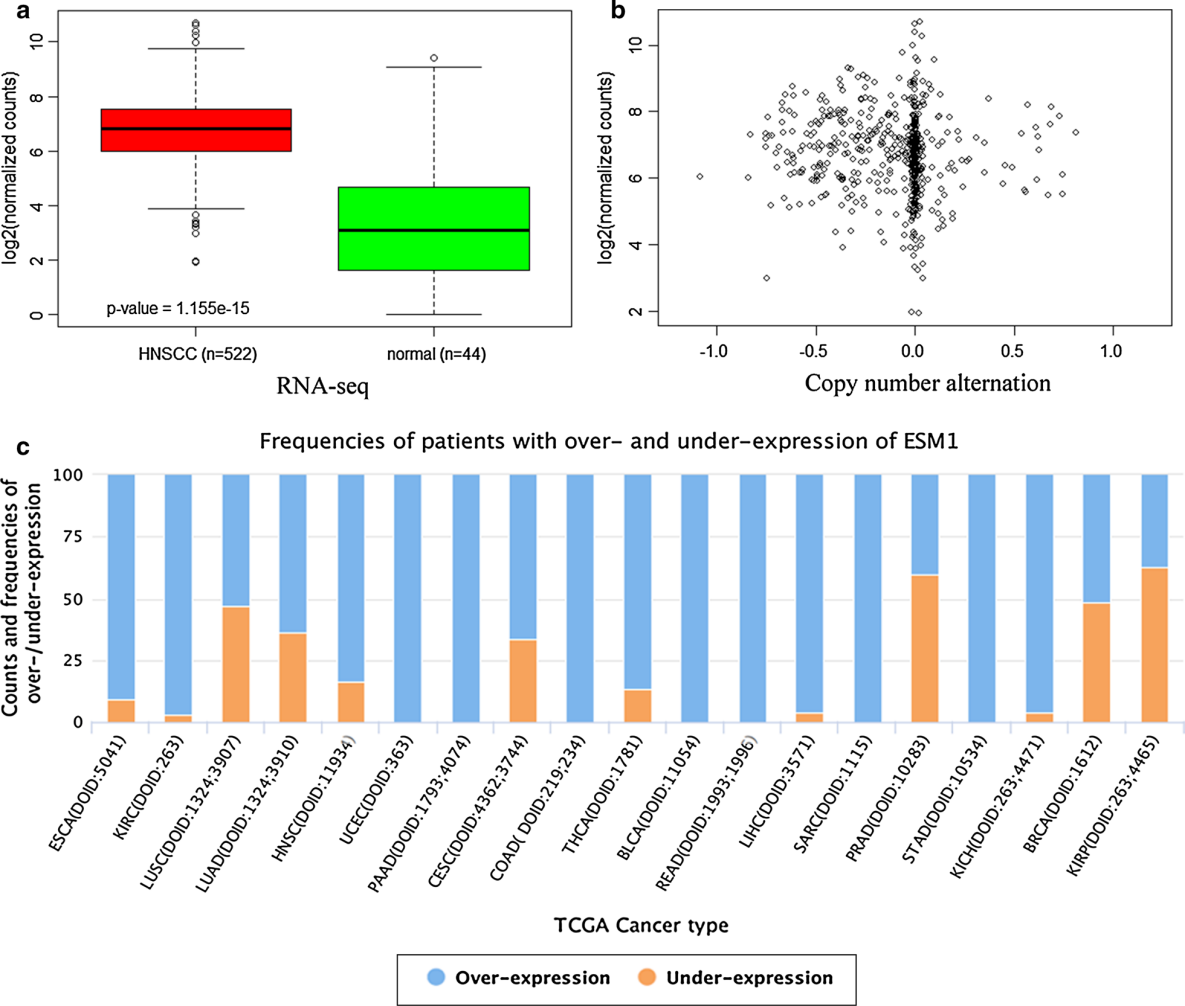
ESM1 is overexpressed in HNSCC from TCGA data. **a** Comparison of expression levels between HNSCC and normal tissues. **b** Copy number does not affect gene expression of ESM1. Positive and negative values indicate gain and loss of copy number, respectively. **c** The BioXpress database reveals that ESM1 is widely overexpressed in human cancers. The frequencies of patients who have an over- (blue) or under- (orange) expression of ESM1 in each cancer type are shown. During paired analysis between cancerous and adjacent tissues, all log2 fold change (log2FC) values greater than zero for ESM1 are considered to be overexpression, less than zero to be under-expression. The abbreviations are as follows: BLCA: urinary bladder cancer; BRCA: breast cancer; CESC: cervical squamous cell carcinoma; COAD: colon adenocarcinoma; ESCA: esophageal cancer; HNSC: head and neck cancer; KICH: kidney chromophobe adenocarcinoma; KIRC: kidney renal clear cell carcinoma; KIRP: kidney papillary renal cell carcinoma; LIHC: liver cancer; LUAD: lung adenocarcinoma; LUSC: lung squamous cell carcinoma; PAAD: pancreas adenocarcinoma; PRAD: prostate cancer; READ: rectum adenocarcinoma; SARC: sarcoma; STAD: stomach cancer; THCA: thyroid cancer; UCEC: uterine cancer

The Cancer Genome Atlas (TCGA) data have become an important and widely used resource in cancer research [29]. As for HNSCC, currently there are 522 cancerous and 44 normal samples that have been sequenced at the RNA level using high-throughput sequencing technology. As shown in Fig. 1a, the RNA-seq revealed that ESM1 was dramatically overexpressed in HNSCC. Because genetic instability such as gene copy number alteration is a general potential factor affecting gene expression in cancers, we therefore also examined the relationship between *ESM1* copy number and gene expression in 514 common HNSCC samples. As shown in Fig. 1b, *ESM1* has frequent heterozygous loss of copy number in HNSCC with a ratio of about 36.97% (193/522) compared to gain of copy number (about 9.39%, 49/522). However, there is no apparent correlation between copy number variation (CNV) and gene expression (Fig. 1b), suggesting that some other mechanisms may control the up-regulated expression of ESM1 in HNSCC. The overexpression of ESM1 in HNSCC and other cancers was also confirmed based on paired analysis of TCGA data (Fig. 1c).

**Fig. 2:**
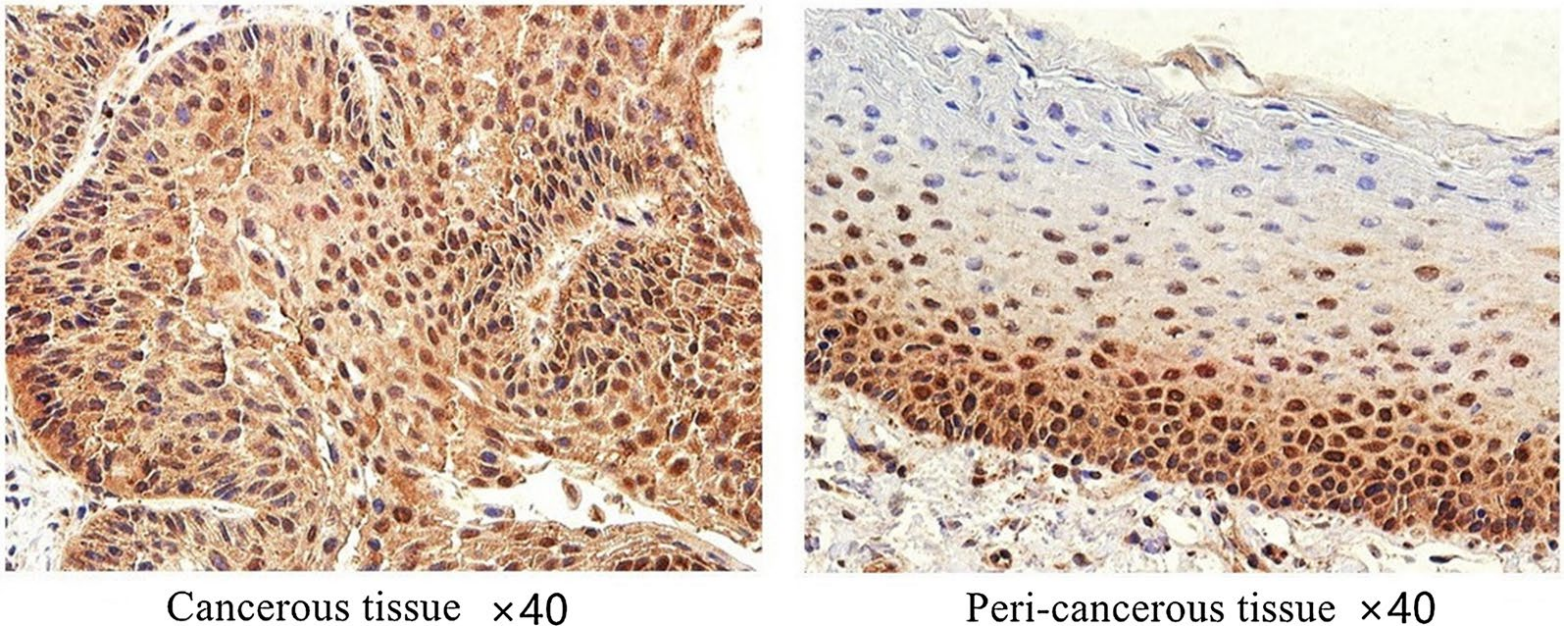
Immunohistochemical analysis shows that ESM1 is overexpressed in HNSCC

Because these results from TCGA data were at the RNA level, we then detected ESM1 expression at the protein level in the 21 laryngeal or hypopharyngeal cancer samples. As shown in Table 1, Fig. 2 and Additional file 1, ESM1 was significantly overexpressed at the protein level in these cancers but there was no apparent correlation with clinical or pathologic stage.

**Table 1:** Correlations between ESM1 expression and clinical features of laryngeal or hypopharyngeal cancer

Clinical factor	Sample size	Correlation coefficient	*p* value
Age	21	− 0.493	0.023
Grade^a^		− 0.138	0.552
1	3		
2	13		
3	5		
Stage^a^		− 0.044	0.850
I	3		
II	5		
III	7		
IV	6		

^a^Grade and stage were referred to the American Joint Committee on Cancer (AJCC) TNM staging classification for laryngeal and hypopharyngeal cancer (7th edition, 2010)

### Identification of *ANGPT2* as the gene most correlated with *ESM1* in HNSCC

**Fig. 3:**
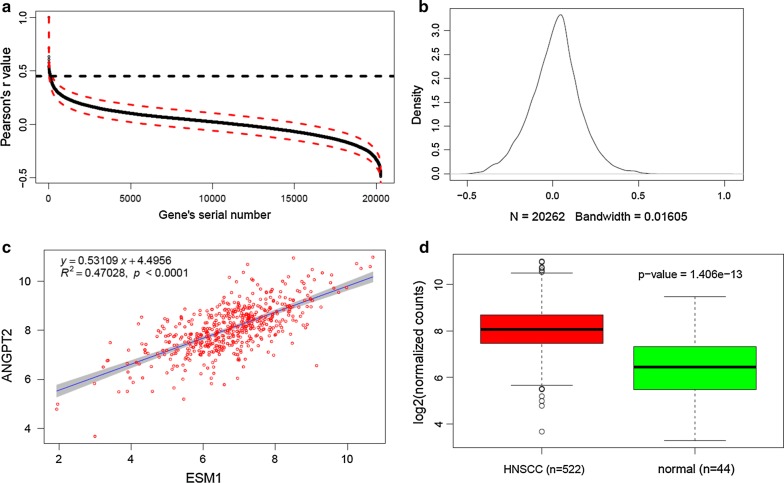
*ANGPT2* is the gene most correlated with *ESM1* and is also overexpressed in HNSCC. **a** The distribution of Pearson correlation coefficients between ESM1 and other genes. The dash lines in red indicate confidence intervals. The black dash line represents a cut-off r value with 0.45. **b** Kernel density distribution of all r values in **a**. **c**. The expressional correlation between ESM1 and ANGPT2, with a linear regression estimation shown. The shade band indicates a 95% confidence interval. **d** ANGPT2 is also overexpressed in HNSCC

Since up-regulation of *ESM1* was not associated with copy number alteration in HNSCC, we next investigated the potential regulatory mechanisms, mainly focusing on transcription factors (TFs). Generally, transcription factor search tools identify potential TF binding sites (TFBSs) by sequence matching, which often results in dozens or even hundreds of candidate TFBSs and thus it is difficult to identify the true transcription factors that have important regulatory roles. Therefore, we first identified the co-expressed genes based on Pearson correlation. In total, there were 85 genes with r ≥ 0.45 and all of these genes were significantly overexpressed in HNSCC based on our analysis (Table 2). Some of them have been reported to be associated with HNSCC. The gene most correlated was *ANGPT2* (angiopoietin 2, also known as Ang-2) with a correlation coefficient (r) of 0.7133 (p value = 3.95E−89) (Fig. 3a), suggesting that a tightly co-regulated mechanism exists between *ESM1* and *ANGPT2*. ANGPT2 was also up-regulated in HNSCC (Fig. 3b, Table 2).

**Table 2:** List of 85 genes co-expressed with ESM1 and with r ≥ 0.45

Gene symbol	Correlation with ESM1	P value*	Adjusted p value*	Status	P value**	Adjusted p value**
ESM1	1.00E+00	0.00E+00	0.00E+00	Up-regulation	1.16E−15	2.52E−14
ANGPT2	7.13E−01	3.95E−89	4.00E−85	Up-regulation	1.41E−13	1.94E−12
COL4A1	6.32E−01	1.63E−64	1.10E−60	Up-regulation	1.28E−21	1.75E−19
COL4A2	6.05E−01	6.72E−58	3.40E−54	Up-regulation	7.94E−20	5.22E−18
SPRY4	5.80E−01	3.88E−52	1.57E−48	Up-regulation	3.66E−18	1.49E−16
LOXL2	5.74E−01	5.31E−51	1.79E−47	Up-regulation	2.00E−22	4.31E−20
APLN	5.53E−01	1.47E−46	4.25E−43	Up-regulation	1.04E−13	1.48E−12
COL10A1	5.45E−01	3.51E−45	8.90E−42	Up-regulation	1.48E−22	3.72E−20
ITGA1	5.41E−01	2.37E−44	5.34E−41	Up-regulation	9.44E−13	1.12E−11
ADAM12	5.40E−01	3.40E−44	6.88E−41	Up-regulation	3.23E−22	6.24E−20
NID2	5.38E−01	9.24E−44	1.70E−40	Up-regulation	7.21E−15	1.31E−13
FLT1	5.37E−01	1.15E−43	1.94E−40	Up-regulation	2.76E−06	9.80E−06
MMP11	5.35E−01	3.88E−43	6.05E−40	Up-regulation	1.52E−24	1.62E−21
BMP8A	5.33E−01	6.04E−43	8.74E−40	Up-regulation	8.76E−24	4.93E−21
DLL4	5.30E−01	2.53E−42	3.42E−39	Up-regulation	5.77E−09	3.33E−08
CTHRC1	5.29E−01	4.40E−42	5.57E−39	Up-regulation	5.26E−19	2.76E−17
STC2	5.24E−01	3.76E−41	4.49E−38	Up-regulation	1.73E−21	2.22E−19
HAPLN1	5.19E−01	2.39E−40	2.69E−37	Up-regulation	1.54E−19	9.23E−18
COL13A1	5.13E−01	2.87E−39	3.06E−36	Up-regulation	6.35E−22	9.97E−20
SOX11	5.11E−01	5.37E−39	5.44E−36	Up-regulation	1.29E−15	2.79E−14
MMD	5.10E−01	7.91E−39	7.63E−36	Up-regulation	5.97E−15	1.11E−13
LEPRE1	5.04E−01	7.37E−38	6.78E−35	Up-regulation	2.31E−21	2.71E−19
COL5A2	5.01E−01	3.05E−37	2.69E−34	Up-regulation	3.63E−20	2.74E−18
ADAMTSL2	5.00E−01	3.78E−37	3.19E−34	Up-regulation	1.19E−16	3.32E−15
LAMA4	4.96E−01	2.00E−36	1.62E−33	Up-regulation	3.53E−07	1.46E−06
CLIC4	4.94E−01	3.11E−36	2.43E−33	Up-regulation	7.44E−13	9.03E−12
P4HA1	4.93E−01	5.24E−36	3.84E−33	Up-regulation	1.32E−17	4.60E−16
EXTL2	4.93E−01	5.30E−36	3.84E−33	Up-regulation	2.34E−09	1.45E−08
GPR4	4.93E−01	5.69E−36	3.95E−33	Up-regulation	1.19E−10	9.38E−10
GJC1	4.93E−01	5.85E−36	3.95E−33	Up-regulation	6.71E−22	1.05E−19
CCDC102B	4.92E−01	9.19E−36	6.01E−33	Up-regulation	6.92E−08	3.28E−07
PLOD1	4.90E−01	1.41E−35	8.68E−33	Up-regulation	7.59E−19	3.80E−17
P4HA3	4.90E−01	1.41E−35	8.68E−33	Up-regulation	4.71E−15	9.05E−14
TMEFF1	4.89E−01	2.22E−35	1.29E−32	Up-regulation	5.29E−18	2.05E−16
C12orf23	4.89E−01	2.43E−35	1.37E−32	Up-regulation	8.80E−09	4.91E−08
SC65	4.88E−01	3.24E−35	1.77E−32	Up-regulation	1.17E−18	5.49E−17
IKBIP	4.88E−01	3.88E−35	2.07E−32	Up-regulation	3.88E−19	2.11E−17
ZNF697	4.86E−01	7.28E−35	3.78E−32	Up-regulation	5.98E−13	7.38E−12
CERCAM	4.85E−01	8.37E−35	4.24E−32	Up-regulation	1.69E−14	2.81E−13
SPARC	4.85E−01	9.04E−35	4.47E−32	Up-regulation	1.52E−14	2.57E−13
PXDN	4.85E−01	1.12E−34	5.38E−32	Up-regulation	2.28E−13	3.03E−12
ACAN	4.84E−01	1.35E−34	6.35E−32	Up-regulation	1.53E−12	1.75E−11
CHN1	4.83E−01	2.04E−34	9.18E−32	Up-regulation	1.93E−16	5.06E−15
IBSP	4.83E−01	2.35E−34	1.02E−31	Up-regulation	4.11E−20	3.01E−18
FNDC3B	4.83E−01	2.38E−34	1.02E−31	Up-regulation	3.99E−14	6.13E−13
THY1	4.82E−01	2.76E−34	1.16E−31	Up-regulation	4.95E−13	6.18E−12
FKBP10	4.81E−01	3.59E−34	1.48E−31	Up-regulation	4.85E−12	5.00E−11
COL1A1	4.80E−01	5.38E−34	2.18E−31	Up-regulation	6.42E−19	3.29E−17
CD276	4.79E−01	9.39E−34	3.73E−31	Up-regulation	5.95E−22	9.68E−20
HEYL	4.78E−01	1.22E−33	4.76E−31	Up-regulation	3.39E−11	2.95E−10
IL11	4.78E−01	1.37E−33	5.25E−31	Up-regulation	1.09E−24	1.38E−21
SERPINH1	4.77E−01	1.43E−33	5.35E−31	Up-regulation	1.85E−22	4.31E−20
GUCY1A2	4.77E−01	1.58E−33	5.80E−31	Up-regulation	1.97E−09	1.25E−08
LAMB1	4.77E−01	1.88E−33	6.81E−31	Up-regulation	5.98E−16	1.41E−14
PDIA5	4.75E−01	2.87E−33	1.00E−30	Up-regulation	3.81E−17	1.17E−15
TTYH3	4.75E−01	2.94E−33	1.01E−30	Up-regulation	6.54E−21	6.76E−19
COL6A1	4.75E−01	3.49E−33	1.18E−30	Up-regulation	4.47E−14	6.78E−13
COL5A1	4.74E−01	4.53E−33	1.50E−30	Up-regulation	5.00E−20	3.57E−18
CALU	4.74E−01	5.09E−33	1.66E−30	Up-regulation	3.39E−14	5.29E−13
KDELR3	4.74E−01	5.45E−33	1.75E−30	Up-regulation	3.66E−08	1.83E−07
COL6A3	4.71E−01	1.34E−32	4.18E−30	Up-regulation	2.98E−14	4.71E−13
EPOR	4.69E−01	2.75E−32	8.44E−30	Up-regulation	2.58E−12	2.82E−11
COL11A1	4.68E−01	3.42E−32	1.03E−29	Up-regulation	1.48E−15	3.16E−14
ENO2	4.67E−01	4.78E−32	1.42E−29	Up-regulation	3.28E−20	2.51E−18
PPAPDC1A	4.67E−01	5.91E−32	1.74E−29	Up-regulation	4.50E−20	3.24E−18
FN1	4.67E−01	6.12E−32	1.77E−29	Up-regulation	2.33E−14	3.78E−13
POSTN	4.66E−01	8.39E−32	2.36E−29	Up-regulation	2.13E−16	5.50E−15
ADAMTS12	4.65E−01	8.94E−32	2.48E−29	Up-regulation	3.76E−14	5.81E−13
NOX4	4.65E−01	1.15E−31	3.10E−29	Up-regulation	1.49E−14	2.52E−13
GLT25D1	4.64E−01	1.30E−31	3.46E−29	Up-regulation	3.17E−26	1.61E−22
ADAMTS7	4.61E−01	3.73E−31	9.69E−29	Up-regulation	1.64E−17	5.55E−16
MFAP2	4.58E−01	9.93E−31	2.55E−28	Up-regulation	3.26E−22	6.24E−20
RCN3	4.57E−01	1.38E−30	3.50E−28	Up-regulation	6.41E−15	1.19E−13
TNFAIP6	4.57E−01	1.57E−30	3.89E−28	Up-regulation	1.36E−07	6.10E−07
C5orf13	4.56E−01	1.77E−30	4.26E−28	Up-regulation	1.84E−13	2.48E−12
VEGFA	4.56E−01	1.92E−30	4.58E−28	Up-regulation	6.16E−09	3.54E−08
COL3A1	4.55E−01	2.53E−30	5.95E−28	Up-regulation	9.35E−16	2.07E−14
TBXA2R	4.55E−01	2.84E−30	6.62E−28	Up-regulation	4.57E−09	2.68E−08
PCDH12	4.55E−01	3.20E−30	7.38E−28	Up-regulation	1.34E−08	7.24E−08
CDR2	4.53E−01	4.71E−30	1.07E−27	Up-regulation	1.71E−11	1.58E−10
PDGFB	4.53E−01	5.51E−30	1.23E−27	Up-regulation	8.78E−07	3.40E−06
ITGA5	4.52E−01	6.49E−30	1.41E−27	Up-regulation	8.03E−19	3.96E−17
FOXS1	4.51E−01	1.06E−29	2.28E−27	Up-regulation	2.77E−16	7.04E−15
PPEF1	4.50E−01	1.31E−29	2.77E−27	Up-regulation	9.05E−23	2.58E−20
IGFBP7	4.50E−01	1.37E−29	2.86E−27	Up-regulation	3.15E−10	2.28E−09

The column ‘Correlation with ESM1’ indicates Pearson correlation coefficients between indicated genes and ESM1. The null hypothesis is no correlation or no differential expression. The functions “cor.test” and “p.adjust” in R software environment are used for p value calculation and p value adjustment (method = “BH”)

*Statistical test for correlation analysis

**Statistical test for differential expression between cancerous and adjacent tissues

**Fig. 4:**
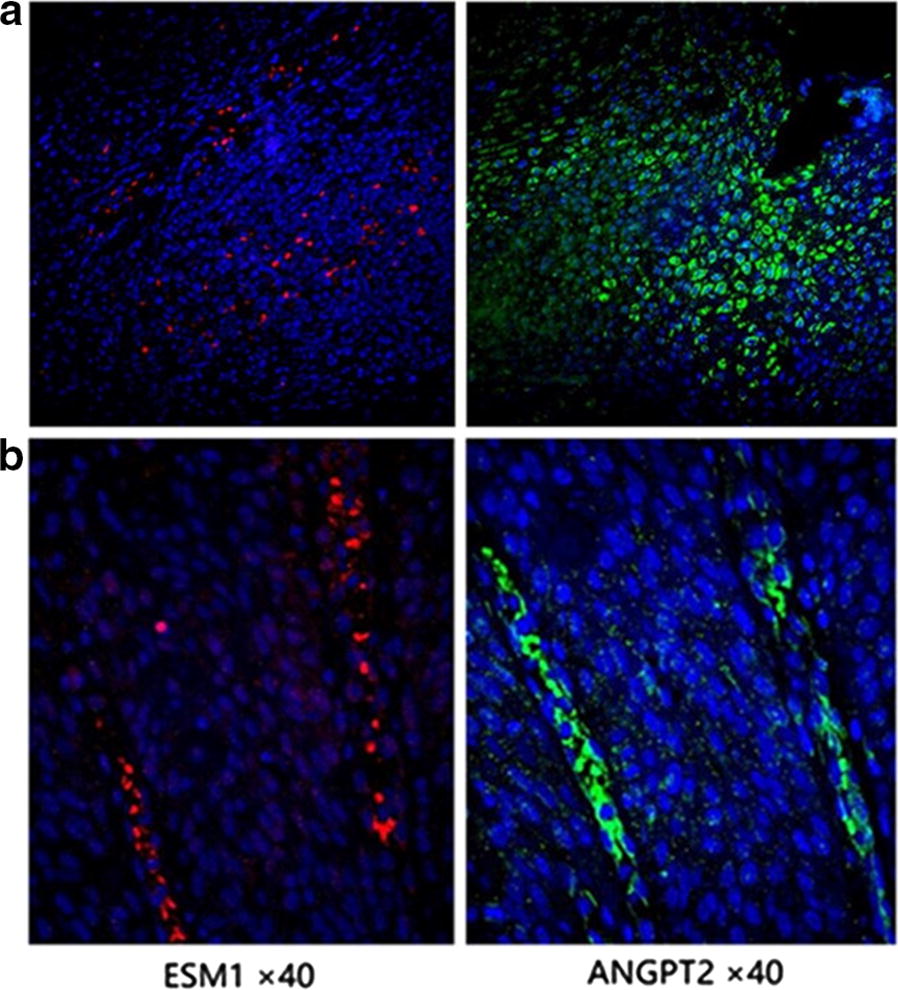
Immunofluorescence staining shows that ESM1 and ANGPT2 both expressed in the same tissues. **a** Both of ANGPT2 and ESM1 can be expressed in cancerous epithelial cells. **b** Similar expression pattern was observed in interstitial tissues

We further confirmed the co-expression of ANGPT2 and ESM1 using immunofluorescence assay. The results showed that both of ESM1 and ANGPT2 could be expressed in the same tissues, either in the cancerous epithelial cells (Fig. 4a) or interstitial tissues (Fig. 4b).

### Identification of AP-1 as an important regulator of ESM1

**Fig. 5:**
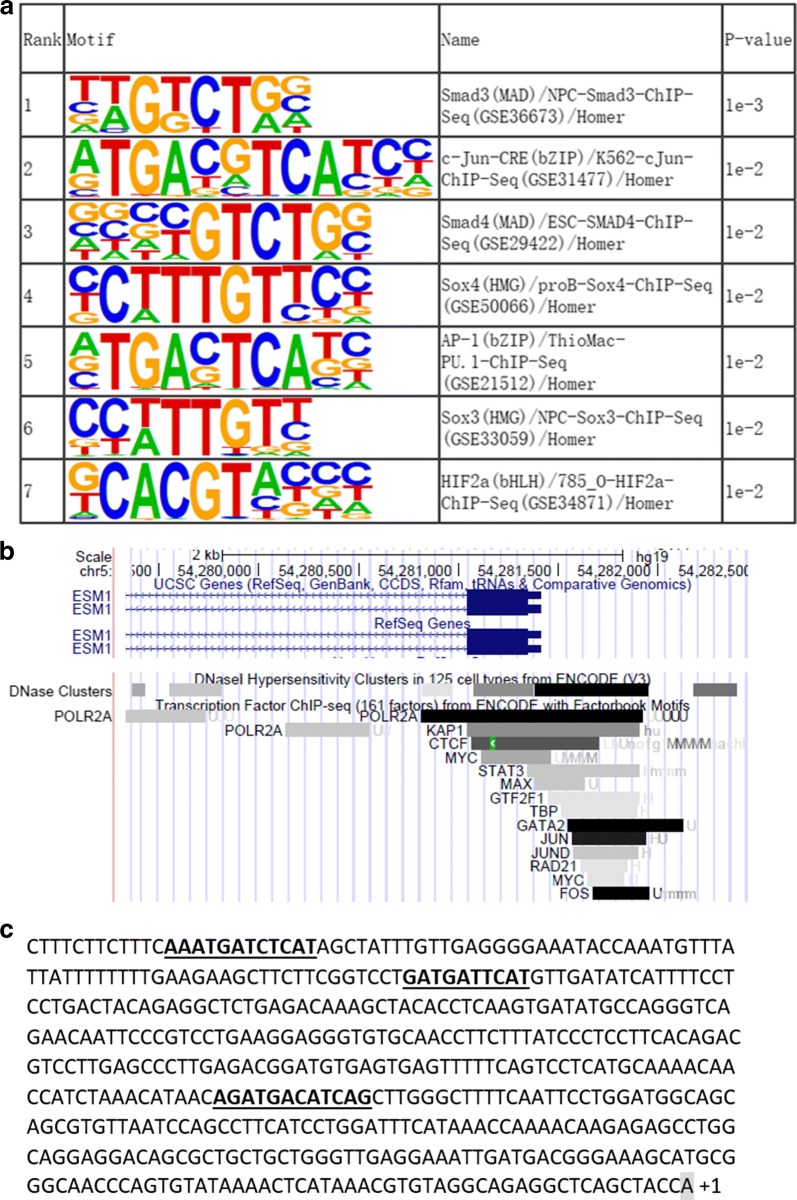
AP-1 is an important regulator of ESM1 expression. **a** Homer known motif enrichment result. **b** Transcription factor ChIP-seq result on UCSC genome browser. In the ChIP-seq track, each block represents a peak bound by the corresponding transcription factor. **c** AP-1 binding sites in the promoter region of ESM1. Sequence 500 bp before the transcription start site (TSS) is shown. The shaded base A indicates TSS (+ 1 position). The underlined bold bases indicate potential AP-1 binding sites matching the second and fifth motifs in the enriched known Homer motifs

Next, we used the Homer program to identify possible enriched motifs in the promoter regions from – 500 to + 100 bp around the transcription start site (TSS) of the 85 correlated genes. As shown in Fig. 5a, seven motifs including Smad3, Smad4, c-Jun, AP-1, Sox3, Sox4 and HIF2α were significantly enriched, suggesting they play important roles in regulating the correlated network of ESM1.

We then used ChIP-seq data from the ENCODE project to filter the results. We found that only AP-1, which is a heterodimer composed of proteins belonging to the c-Fos, c-Jun, ATF and JDP families, overlapped in the promoter region of ESM1 (Fig. 5b). We also observed that AP-1 or its subunit binding sites exist in the promoter region (Fig. 5c). These results further confirmed that AP-1 is an important regulator of ESM1.

## Discussion

Endocan is a 50 kDa soluble proteoglycan secreted by vascular endothelial cells, especially from the inflamed endothelium, thereby it is also thought to play a role in the pathogenesis of vascular disorders, inflammation and endothelium dysfunction [9]. It can bind to the leukocyte integrin LFA-1 (CD11a/CD18), and prevents the specific binding of ICAM-1 to LFA-1, and may therefore influence both the recruitment of circulating lymphocytes to inflammatory sites and LFA-1-dependent leukocyte adhesion and activation [34]. Endocan is clearly overexpressed in many cancers and has also been shown to be directly involved in tumor progression as observed in mouse models of human tumor xenografts [9]. In the current study, we have confirmed that endocan is also dramatically overexpressed in HNSCC. A recent study revealed that ESM1 could mediate nerve growth factor receptor (NGFR)-induced invasion and metastasis in murine oral squamous cell carcinoma [35]. All these results indicate that ESM1 may be a potential therapeutic target in HNSCC.

An early study showed that Ets-binding motifs were mainly responsible for endothelial-cell-specific expression of ESM1 in vitro, though putative binding sites for GATA, AP1, AP4, NF1, and CREB/ATF transcription factors were also speculated [36]. We also investigated the regulatory mechanism using publically available data and found that AP-1 may be a key regulator of ESM1, particularly for the co-expressed network centered on ESM1. ESM1 can be activated by inflammation, cytokines and vascular growth factors, and in fact, AP-1 activity is also regulated by a broad range of physiological and pathological stimuli, including cytokines, growth factors, stress signals and infections, as well as oncogenic stimuli [37]. AP-1 mediates regulation involved in many biological processes such as proliferation, differentiation, apoptosis and transformation. A typical upstream signal pathway for activation of AP-1 that has been widely studied is the Ras-MAPK-ERK pathway, which is one of several important pathways for targeting therapy in HNSCC [38].

Besides AP-1, ChIP-seq from the ENCODE project also suggests that other transcription factors such as STAT3 (signal transducer and activator of transcription 3), TBP (TATA-box binding protein), GATA2 (GATA binding protein 2), RAD21 (RAD21 cohesin complex component) and MYC (MYC proto-oncogene, bHLH transcription factor) are also potential regulators of ESM1. Considering the genes co-expressed with ESM1, AP-1 probably plays a key role but other factors may synergize the regulation. Further details still need investigation.

We identified the genes co-expressed with ESM1 in HNSCC and the most correlated gene is *ANGPT2*. ANGPT2 can also be regulated by Ets-1 and AP-1 [39, 40], further confirming their correlation. As shown in Fig. 4, although the expressional patterns of ESM1 and ANGPT2 are not fully overlapped, co-expression in some of the same cells can be truly observed. However, relatively lower immunofluorescence positivity for ESM1 in Fig. 4 was observed as compared with the DAB positivity pattern in Fig. 2. This may be due to different specimen and antibodies used in two assays. On the other hand, correlation doesn’t mean co-expression in the same cells when bulk RNA-seq data were used, they can be expressed in different cell types but could also show positive correlation. A recent study shows ANGPT2 can be regulated by the synaptic protein neuroligin 2 (NLGN2) [41], whether ESM1 is also regulated by NLGN2 needs further investigation. Angiopoietins, including ANGPT1, ANGPT2, ANGPT3 and ANGPT4, are vascular growth factors that control microvascular permeability, vasodilation, and vasoconstriction by signaling smooth muscle cells. Antiangiogenic agents can normalize the tumor microenvironment, combining antiangiogenic therapies with immune-checkpoint inhibitors potentially improve patient outcomes for the treatment of a range of solid tumors [42].

ANGPT1 is critical for vessel maturation, adhesion, migration, and survival, but ANGPT2 is an antagonist of ANGPT1 promoting cell death and disrupting vascularization; [43] however, VEGF and ANGPT2 appear to play crucial roles in the balance between vascular regression and growth of this subset of tumors, and the combination can promote neo-vascularization. [42, 44] Mice deficient in ANGPT2 have abnormalities in the blood and lymphatic vasculatures, and also show deficits in rapid leukocyte recruitment to sites of inflammation [45]. This function is very similar to ESM1; however, whether ESM1 and ANGPT2 can be mutually regulated still awaits further investigation.

## Conclusions

In conclusion, we have identified that ESM1 is overexpressed in HNSCC and investigated the regulatory mechanism of ESM1-centered co-expression. These results provide important functional clues for ESM1 dysregulation and regulation in cancers.

## Additional file

**Additional file 1.** ESM1 expression of 21 paired samples of HNSCC with clinical and pathological features.

## Abbreviations

ESM1: endothelial cell specific molecule-1
HNSCC: head and neck squamous cell carcinoma
TCGA: The Cancer Genome Atlas
ANGPT2: angiopoietin-2
ENCODE: Encyclopedia of DNA Elements
HPV: human papilloma virus
TUBB3: βIII-tubulin
HOX: homeobox gene family
MMPs: metalloproteinases
TNF-α: tumor necrosis factor-α
IL: interleukin
VEGF: vascular endothelial growth factor
UCSC: University of California, Santa Cruz
HRP: horseradish peroxidase
DAB: diaminobenzidine
HOMER: Hypergeometric Optimization of Motif EnRichment
TSS: transcription start site
CNV: copy number variation
TFBSs: TF binding sites
NGFR: nerve growth factor receptor
STAT3: signal transducer and activator of transcription 3
TBP: TATA-box binding protein
GATA2: GATA binding protein 2
